# Potential for Electric Vehicle Adoption to Mitigate Extreme Air Quality Events in China

**DOI:** 10.1029/2020ef001788

**Published:** 2021-02-12

**Authors:** J. L. Schnell, D. R. Peters, D. C. Wong, X. Lu, H. Guo, H. Zhang, P. L. Kinney, D. E. Horton

**Affiliations:** 1 Department of Earth and Planetary Sciences and Institute for Sustainability and Energy at Northwestern University, Evanston, IL, USA; 2 now at: Cooperative Institute for Research in Environmental Sciences at the University of Colorado Boulder NOAA/Global Systems Laboratory, Boulder, CO, USA; 3 Program in Environmental Sciences, Northwestern University, Evanston, IL, USA; 4 Environmental Defense Fund, Austin, TX, USA; 5 US Environmental Protection Agency, Research Triangle Park, NC, USA; 6 School of Environment, State Key Joint Laboratory of Environment Simulation and Pollution Control, Tsinghua University, Beijing, China; 7 Department of Earth System Science, University of California Irvine, Irvine, CA, USA; 8 Department of Environmental Science and Engineering, Fudan University, Shanghai, China; 9 Department of Environmental Health, Boston University School of Public Health, Boston, MA, USA

## Abstract

Electric vehicle (EV) adoption promises potential air pollutant and greenhouse gas (GHG) reduction co-benefits. As such, China has aggressively incentivized EV adoption, however much remains unknown with regard to EVs’ mitigation potential, including optimal vehicle type prioritization, power generation contingencies, effects of Clean Air regulations, and the ability of EVs to reduce acute impacts of extreme air quality events. Here, we present a suite of scenarios with a chemistry transport model that assess the potential co-benefits of EVs during an extreme winter air quality event. We find that regardless of power generation source, heavy-duty vehicle (HDV) electrification consistently improves air quality in terms of NO_2_ and fine particulate matter (PM_2.5_), potentially avoiding 562 deaths due to acute pollutant exposure during the infamous January 2013 pollution episode (~1% of total premature mortality). However, HDV electrification does not reduce GHG emissions without enhanced emission-free electricity generation. In contrast, due to differing emission profiles, light-duty vehicle (LDV) electrification in China consistently reduces GHG emissions (~2 Mt CO_2_), but results in fewer air quality and human health improvements (145 avoided deaths). The calculated economic impacts for human health endpoints and CO_2_ reductions for LDV electrification are nearly double those of HDV electrification in present-day (155M vs. 87M US$), but are within ~25% when enhanced emission-free generation is used to power them. Overall, we find only a modest benefit for EVs to ameliorate severe wintertime pollution events, and that continued emission reductions in the power generation sector will have the greatest human health and economic benefits.

## Plain Language Summary

Electric vehicles (EVs) offer potential air quality and climate change co-benefits, but due to varying power generation and vehicle types, and because air pollution chemistry is nonlinear, it is not clear to what extent EVs could provide mediation, especially during extreme air pollution episodes. China is both rapidly adopting EVs and frequently experiences poor air quality. We use an air quality model that simulates the complex interplay between weather and air quality to examine the potential co-benefits of EVs in China during a historical pollution episode. We simulate both light- and heavy-duty vehicle adoption to show their individual benefits, and demonstrate the need for low-emission electricity generation to maximize co-benefits. Overall, we find that heavy-duty fleet electrification consistently improves air quality and reduces mortality, but offers little climate change benefits without enhanced emission-free electricity generation. Light-duty vehicles, however, offer large climate change benefits but few air quality improvements, highlighting the need for cross-modal adoption strategies.

## 1. Introduction

China faces the concurrent challenges of mitigating anthropogenic climate change and improving air quality. China contributes ~30% of global CO_2_ emissions in 2014 (Boden et al., 2017) and ambient pollution accounts for ~17% of its annual deaths (Rohde et al., 2015). Mitigation strategies that simultaneously target both challenges, such as the electrification of the transportation sector, are desirable and needed (Haines, 2017; Patz, 2020). China’s transportation sector contributes ~9% of its total CO_2_ emissions (Zheng et al., 2018) and is responsible for 100,000+ annual air pollution related premature deaths (Anenberg et al.,). While electric vehicles (EVs) remove on-road CO_2_ and tailpipe pollutant emissions and precursors, electricity demands increase emissions from fossil fuel-based electricity generating units (EGUs), which comprise 63% of China’s grid mix in 2016 (IEA, 2017).

Recent studies suggest that extreme pollution episodes will constitute a disparate share of China’s future increases in air quality-related mortality (Hong et al., 2019). Additionally, some have suggested the underlying meteorological conditions of the formation and persistence of extreme events (L.Zhang et al., 2015) have increased in likelihood due to anthropogenic climate change (Callahan et al., 2019; Cai et al., 2017; Zou et al., 2017, 2020), though others suggest the link is uncertain (Shen et al., 2018; Z. Xu et al., 2020). One such extreme pollution episode occurred in January 2013, when over 600M people across China were exposed to extremely high levels of fine particulate matter (PM_2.5_) during a series of pollution episodes (Sheehan et al., 2014). Conditions in Beijing were particularly dire: visibility was reduced to <1 km (Sun et al., 2014), emergency room visits increased ~30% (Ferrreri et al., 2018), and ~690 premature deaths occurred with health impacts totaling 250M+ US$ (Gao et al., 2015). These episodes—often referred to as Airpocalypse in popular media (Beech, 2013; Kaiman, 2013)—motivated significant pollution control efforts in the transportation and energy sectors (Zhang et al., 2019), including a strong regulatory push toward “New Energy Vehicles” like EVs (Reuters, 2020).

A simple accounting of the displacement of on-road to EGU-based emissions can be used to quantify net CO_2_ changes due to EV adoption (e.g., Huo et al., 2015; Peters et al., 2020), but pollutant emission changes are heterogeneous in space and time, and the efficacy of emissions to produce pollution depends on numerous complicating nonlinear chemical and meteorological factors—unlike spatially well-mixed and nonreactive CO_2_. Therefore, efforts to evaluate air quality impacts of EV adoption must use a chemistry-transport model (CTM) to capture complexities of air pollution chemistry, transport, and timing. CTM-based analyses of EV adoption in China are limited despite growing widespread deployment (e.g., He et al., 2018). Moreover, comparisons are challenging due to methodological differences, and key findings can diverge. For example, Peng et al. (2018) found that coal-intensive (75%) electrification of 30% of on-road vehicles does not reduce GHG emissions but could avoid 41k+ deaths, while Liang et al. (2019) found that 27% EV adoption could reduce GHG emissions and avoid 17k+ premature deaths. Both studies simulate electrification of multiple modal types, that is, light-duty vehicles (LDVs) and heavy-duty vehicles (HDVs), which prevents disentangling each mode’s co-benefits. Indeed, the impact of electrifying one mode could mask impacts from others. For example, Huo et al. (2015) used an emission accounting approach and found that in contrast to Peng et al. (2018), electrification of only LDVs could reduce GHG emissions even under coal-intensive electrification.

To clarify benefits and tradeoffs of EV adoption in China, we focus on each mode’s potential to reduce CO_2_ emissions and mitigate extreme winter pollution events. We utilize open-source data and an emission remapping algorithm (Schnell et al., 2019) to estimate changes that result from different EV scenarios (Table 1). To constrain differing emission profile impacts of modal choice we independently assess replacement of equal electricity-demand fractions of China’s HDV and LDV fleets (i.e., 40%). We use a regional chemistry-climate model and quantify changes in CO_2_ and air pollutants from a baseline simulation to each EV scenario. Public health impacts and costs are calculated across seven health endpoints (Gao et al., 2015) caused by acute PM_2.5_ and NO_2_ exposure, which we compare to monetary consequences of CO_2_ emission changes. Further experiments investigate EGU emission rate sensitivities, potential co-benefits of renewable energy adoption, and consequences of coal-only power generation. EV adoption scenarios are simulated using meteorological conditions from January 2013 to assess the potential for air quality remediation during an extreme pollution episode.

## 2. Materials and Methods

### 2.1. Electric Vehicle Adoption Experiments

**Figure 1.**
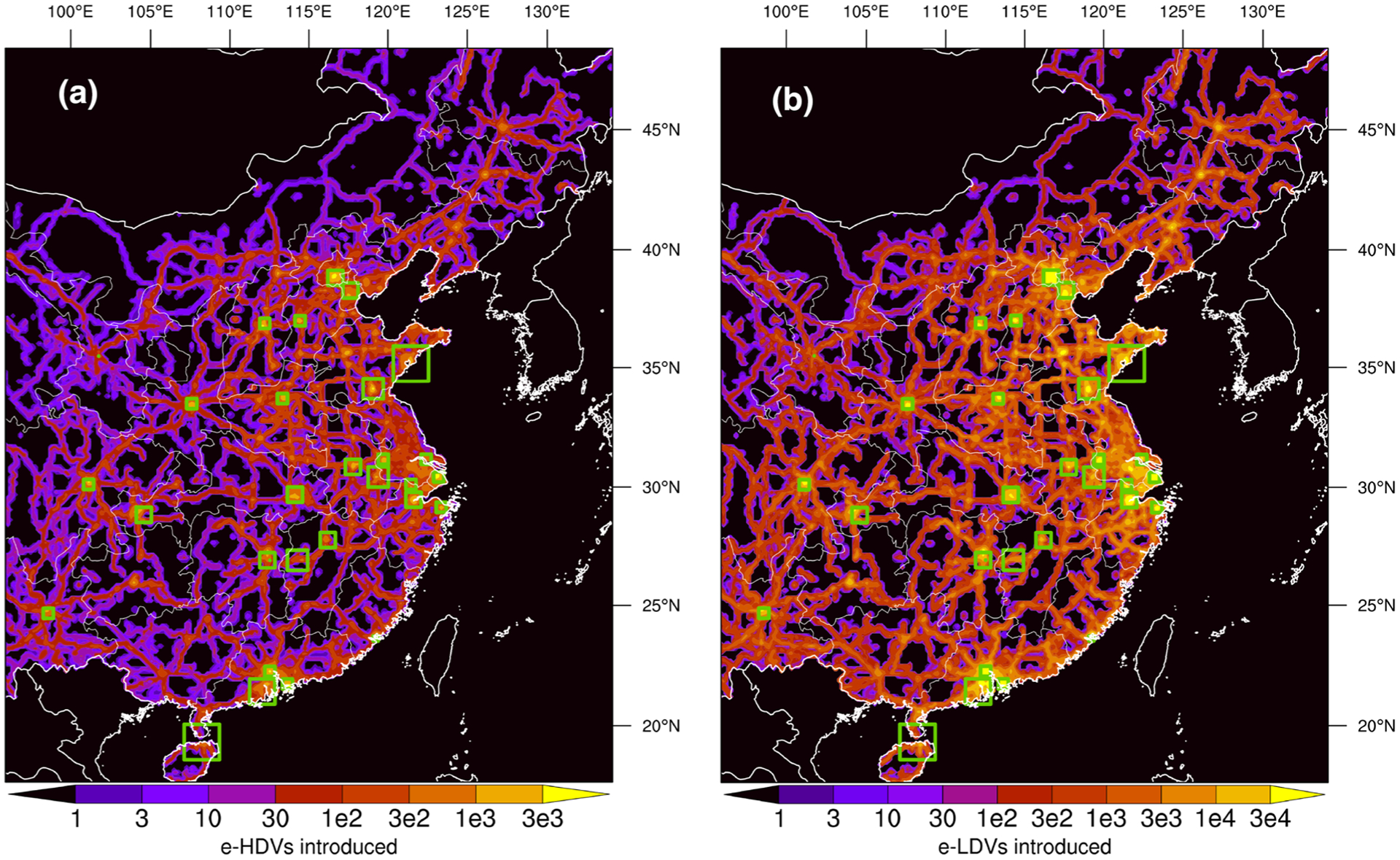
Number of electric vehicles introduced at each 12 km grid cell. (a) e-HDV, (b) e-LDV. EV forward cities (see Materials and Methods) are shown in green. EV, Electric vehicle; HDV, heavy-duty vehicle; LDV, light-duty vehicle.

Each simulation is run from December 22, 2012 to January 31, 2013, with the first 10 days discarded as model spin-up. Our control simulation is referred to as BASE. Our primary electrification (HDV_2015) experiment replaces a total of 1.5M HDVs (~40% of the fleet), with ~33% of these HDVs placed in cities from He et al. (2018); hence, “EV-forward cities” (Figure 1). It is not clear when 40% HDV fleet electrification could be attained, though China has plans for 400K e-HDVs by 2020 (IEA, 2018) and Peng et al. (2018) simulate 30% by 2030; in any case, our scenarios are not meant to be representative of any particular policy pathway. We assume an average operating efficiency of 1.3 kWh km^−1^, similar to the specifications of an electric bus or truck (e.g., https://www.nrel.gov/docs/fy16osti/65274.pdf; https://www.tesla.com/semi). The electricity sector emission rates reflect those from the National Bureau of Statistics (2015). To highlight the impact of recent EGU emission reductions, we perform an experiment (HDV_2010) using emission rates for coal-fired EGUs set to 2010 levels (Liu et al., 2015), as well as an experiment that only uses these coal-fired EGUs (HDV_COAL). We also simulate a scenario (HDV_REN) in which 50% of the marginal electric demand to charge the EVs comes from emission-free sources (e.g., wind, water, and solar). For reference, these sources are projected to make by ~40% of the grid mix by 2020 (IEA, 2017). Emission rates for all generation types except coal-fired EGUs remain the same as in HDV_2015 throughout other experiments.

We compare the co-benefits of e-HDV versus e-LDV adoption by using the total electricity demand from the HDV experiments to instead electrify a fleet of LDVs. The equivalent of each HDV* experiment is also performed for LDVs. For e-LDVs, we use operating efficiencies of 0.16 kWh km^−1^, which represents a new compact EV (e.g., 2019 Tesla Model 3; https://www.fueleconomy.gov/feg/evsbs.shtml); these parameters lead to an equivalent LDV adoption of 39.2M vehicles (coincidently, like HDV, ~40% of the fleet; Figure 1b). To capture a greater uncertainty range for changes in CO_2_ emissions, we compare results using a battery efficiencies for e-LDVs of 0.12 and 0.18 kWh km^−1^ (Huo et al., 2015), and use the same relative scaling for e-HDVs (i.e., 0.975 and 1.4625 kWh km^−1^). Although the total electricity demand is the same between e-HDV and e-LDV experiments, the spatial distribution of the demand differs slightly due to differing intra- and inter-province fleet distributions. In general, LDVs are more concentrated in the most economically developed regions (Figure S1); that is, the national capital region of Beijing-Tianjin-Hebei (BTH), the Yangtze River Delta (YRD: Shanghai, Zhejiang, and Jiangsu), and the Pearl River Delta (PRD: Guangdong). In addition to January 2013, we also simulate HDV_2015 and LDV_2015 for a relatively “clean” month (January 2014) to compare EV-impacts for an extreme episode month to a “normal” month.

### 2.2. Health Impact and Monetary Value Calculations

We calculate the acute health impacts and economic losses that result from surface PM_2.5_ and NO_2_ exposure over the January 2013 episode following the methods of Gao et al. (2015), who apply a Poisson regression model (Guttikunda & Goel, 2013) to estimate the number of cases of mortality and morbidity over seven health endpoints, including premature mortality, respiratory and cardiovascular hospital admissions, outpatient visits (ages 0–14 and 14+), bronchitis, and asthma (Table S1). The number of cases (*ΔE*) is estimated as Equation 1:

(1) $$\Delta E=\overset{\text{\#}\;\text{grids}}{\underset{i=1}{\sum }}\;\Delta POP*IR*\left(1-\frac{1}{e^{\left(\beta \Delta C\right)}}\right)$$

where Δ*POP* is the population exposed to the incremental concentration Δ*C* in the 12 km model grid cell *i*, *IR* is the incidence rate of the health endpoints, and *β* is the concentration-response function. For NO_2_, the only health endpoint we calculate is premature mortalty and our *β* values come from Chen et al. (2018). For PM_2.5_, we use updated, more conservative *β* values from Chen et al. (2017) for all-cause mortality, but apply the same input data and parameters as Gao et al. (2015) in our calculations for other health endpoints: we use the Gridded Population of the World v4 for the year 2015 for population data (https://sedac.ciesin.columbia.edu/data/collection/gpw-v4) and *β* and *IR*s are from a range of sources (Table S1). The *β* values represent the increase in daily mortality and morbidity cases due to a 10 μg m^−3^ increase in two-day average PM_2.5_ or NO_2_ and the *IR*s were converted from an annual to a daily value assuming cases are equally distributed. Like Gao et al. (2015), we also use the WHO 24-h average PM_2.5_ guideline value of 25 μg m^−3^ to obtain the incremental concentration Δ*C*; that is, we assume no health impacts are incurred below this value. For NO_2_ we use a reference value of zero. We calculate the monetary value associated with each health endpoint using the unit loss values from Table 2 of Gao et al. (2015), which are taken from Huang and Zhang (2013). We note, however, the monetary valuation depends on methodology, data source, and the exposure-response functions used to calculate impacts (Li et al., 2018). To calculate the avoided (or added) health and economic impacts due to fleet electrification, we subtract the impacts of the sensitivity simulation from the impacts calculated for BASE.

### 2.3. Air Quality Model Description

Our experiments use the two-way coupled Weather Research and Forecasting (WRF, v3.8; Skamarock et al., 2008) and Community Multi-scale Air Quality (CMAQ, v5.2; Byun et al., 2006) modeling system (WRF-CMAQ; Wong et al., 2012). WRF is run with 30 vertical levels from the surface to 50 hPa at 12 km horizontal resolution extending from 17.6°S–49.6°N and 95.8°E–134.2°E (244 × 294 grid cells). The lowest model layer is ~30 m thick, with the first ~7 layers in the bottom 1 km. Initial and time-varying boundary conditions are provided by the NCEP FNL Operational Model Global Tropospheric Analyses dataset (https://rda.ucar.edu/datasets/ds083.2/). The model is run with analysis nudging above the boundary layer using Four Dimensional Data Assimilation (FDDA) with nudging coefficients of 3.0 × 10^−4^ s^−1^ for temperature and winds and 1.0 × 10^−4^ s^−1^ for water vapor mixing ratio. The model physics options include the Morrison 2-moment microphysics scheme (Morrison et al., 2009), version 2 of the Kain-Fritsch (KF2) cumulus cloud parameterization (Kain, 2004), the Asymmetric Convective Model version 2 (ACM2) for the planetary boundary layer (Pleim, 2007a, 2007b), and the Pleim-Xiu land surface model (Xiu & Pleim, 2001) with soil moisture nudging (Pleim & Gilliam, 2009; Pleim & Xiu, 2003) during the 10-day spin-up period. We use the Rapid Radiative Transfer Model for GCMs (RRTMG) for both our shortwave and longwave radiation schemes, for which the two-way model has been developed to use. WRF is run with a 60 s time step and a 20 min radiation time step. CMAQ is run with the CB05 gas phase mechanism with version 6 of the aerosol module (AERO6) and aqueous/cloud chemistry. CMAQ is coupled to WRF at a frequency of 1:5 (i.e., CMAQ is run every 5 min). Sensitivity tests over our domain show only small differences in simulated PM_2.5_ abundances for higher frequency coupling. Initial and time-varying chemical boundary conditions are from MOZART-4/GEOS5 (https://www.acom.ucar.edu/wrf-chem/mozart.shtml).

Anthropogenic emissions were generated with raw inputs from EDGAR version 4.3.2 (http://edgar.jrc.ec.europa.eu/overview.php?v=432_AP, last access April 10, 2020) using the methods of Wang et al. (2014). Primary PM and VOCs are speciated to model species based on the SPECIATE 4.2 database (Hsu & Divita, 2008). Biogenic emissions are generated using the Model of Emissions of Gases and Aerosols from Nature (MEGAN) version 2.10 (Guenther et al., 2006), while open burning emissions are generated based on the Fire Inventory from NCAR (Wiedinmyer et al., 2011). Emissions of dust and sea salt are calculated online. Although the EDGAR emissions represent year 2010, total Chinese emissions in 2013 are similar (Zheng et al., 2018). In general, transportation emissions increased and power sector emission decreased over the 2010–2013 time period. Onroad and power sector emissions were processed separately and merged after modifications for individual scenarios. The premerged processed emissions that exclude onroad and power sectors were anomalously high in some grid cells, which compounded PM_2.5_ simulation biases. To remedy these biases, we smoothed the 50 largest anomalous values of each emitted species in each emission layer prior to merging with the unmodified onroad and power sector emissions. Anomalous values were smoothed by averaging the eight neighboring grid cells. Grid cell smoothing sensitivity tests were performed until a near-zero mean bias over Beijing was attained.

### 2.4. Model Evalution

Figure S2 compares the time series of WRF-CMAQ simulated daily averaged surface temperature, relative humidity, and 10 m wind speed as compared to NOAA National Centers for Environmental Prediction Integrated Surface Database (https://www.ncdc.noaa.gov/isd/data-access). Our comparisons are with observations sites closest to the U.S. Embassy locations that measure PM_2.5_. Overall, the model performs very well for these variables at these locations. WRF generally underestimates surface temperatures (mean bias [MB] = −0.4 to −1.5) but matches daily variability well—correlations (*r*) range from 0.85 to 0.97. Relative humidity performance is good over Beijing (MB = −3%, *r* = 0.84), though over Chengdu, WRF is biased low by over 20% (*r* = 0.66). Wind speed is also simulated well, with MBs ranging from −1.2 to 0.2 m s^−1^ and high correlations, particularly over Shanghai and Guangzhou.

Figure S3 shows the hourly and daily averaged PM_2.5_ time series for WRF-CMAQ as compared to surface observations from United States Embassy sites in Beijing, Shanghai, Guangzhou, and Chengdu (http://www.stateair.net/web/historical/1/1.html). The model is biased high over three of the four locations, ranging from −0.7 μg m^−3^ (−0.4%) over Beijing to 88 μg m^−3^ (106%) over Guangzhou. The lowest (highest) bias generally occurs during midday (evening) when PM_2.5_ is at a minimum (maximum). Comparing the observed timeseries to the average time series of the nine grid cells around the observation site reveals extremely pronounced spatial variability that the emissions or model may not appropriately delineate. For example, Beijing’s bias decreases from −0.7 to −69 μg m^−3^; Shanghai from 65 to 21 μg m^−3^; Guangzhou from 88 to 67 μg m^−3^; and Chengdu from 33 to −8.4 μg m^−3^. Over Beijing, Shanghai, and Chengdu, WRF-CMAQ matches both the hourly (Pearson correlation, *r*_hour_ = 0.51–0.74) and daily (*r*_day_ = 0.64–0.88) variability of PM_2.5_ well, but it performs poorly over Guangzhou (*r*_day_ = 0.21). Comparisons with Guangzhou’s adjacent grid cells yield similarly poor agreement. We attempted to remedy the poor performance in the vicinity of Guangzhou by testing several WRF physics options (e.g., cumulus physics, stronger nudging and/or nudging in the boundary layer, number of vertical layers, time step(s), etc.). Using stronger nudging coefficients within the boundary layer and at the surface slightly improved the performance over Guangzhou in terms of matching daily variability, but doing so increased the bias in the four cities substantially, and so we retained our original parameters. We also perform a sensitivity simulation without the aerosol-radiation feedback, which reduces PM_2.5_ concentrations (and thus decreases the bias at three of the four sites), but it decreases the correlation at each site (orange lines in Figure S3). On the final two days of our simulation (January 30–31), we observe a substantial high bias in simulated PM_2.5_ over Beijing, which accounts for nearly 30% of the total monthly deaths. This bias occurs in the model whether the feedback is included or not. We suspect this bias results partly from too little vertical diffusion common during the shallow stable boundary layer that occurred during the period and that coincided with other conditions conducive to pollutant accumulation (i.e., high humidity and low wind speeds; Figure S2).

### 2.5. Emission Remapping

We construct our vehicle electrification emission datasets using the methods described in Schnell et al. (2019). We slightly modify the methods due to differences in data sources and modeling system. Our electrification emissions (E*) are calculated as Equation 2:

(2) $$E_{s,t,j}^{*}=E_{s,t,j}^{0}-E_{s,t,j}^{ICE}+E_{s,t,j}^{EGU}$$

where $E_{s,t,j}^{0}$ is the unmodified CMAQ-ready emissions (i.e., hourly, on the 12 km grid, and speciated to the chemical mechanism) for species *s* at hour *t* and grid cell $x_{j},E_{s,t,j}^{ICE}$ are the emissions associated with conventional internal combustion engine vehicles (ICEVs) transitioned to EVs, and $E_{s,t,j}^{EGU}$ is the emissions from electric generating units (EGUs) that power the added EVs.

#### 2.5.1. Emissions of Replaced Internal Combustion Vehicles

We calculate the emissions of the replaced ICEVs as:

(3) $$E_{s,t,j,m}^{\text{ICEV}}=\overset{M}{\underset{m=1}{\sum }}\;fEV_{j,m}\cdot fE_{s,j,m}^{\text{ICEV}}\cdot E_{s,t,j}^{ONR}+\left(r_{TW}-1\right)\;E_{s,j,m}^{TW}+\left(r_{RW}-1\right)\;E_{s,j,m}^{RW}+\left(r_{BW}-1\right)\;E_{s,j,m}^{BW}$$

where *fEV*_*j,m*_ is the fraction of the ICE vehicles in grid cell *j* and mode *m* converted to EVs, $fE_{s,j,m}^{\text{ICEV}}$ is the fraction of on-road transportation emissions from mode *m*, $E_{s,t,j}^{ONR}$ is the total on-road emissions, and $r_{TW}E_{s,j,m}^{TW}$, $r_{RW}E_{s,j,m}^{RW}$, and $r_{BW}E_{s,j,m}^{BW}$ are respectively the scaled non-exhaust emissions of tire wear, road wear, and brake wear. For $fE_{s,j,m}^{\text{ICEV}}$, we use province-level data from the GAINS model that is linearly interpolated to 2013 using 2010 and 2015 data. To calculate *fEV*_*j,m*_, we first determine the number of vehicles of each mode in each grid cell using GAINS vehicle fleet counts, which we map onto our 12 km grid using the on-road emissions of NO_x_ (NO + NO_2_) as weights for HDVs; for LDVs, we use CO. We then choose the total number of ICEVs to transition and distribute them accordingly. First, we distribute a fraction of the total EVs to the 30 cities that collectively represent over 80% of the EVs in 2015 (He et al., 2018) using their battery EV market size as a weight. To determine in which grid cells those EVs are placed, we choose the smallest box around the city center (i.e., 1, 9, 25, etc.) such that 100% of the ICEVs in the center grid cell can be replaced and no more than 75% in the surrounding cells. This method leads to an unrealistic EV adoption “footprint” for the city of Lanzhou, so we do not simulate enhanced EV adoption there. Also, due to the near-overlapping proximity of Xiangtan and Zhuzhou, we combine them into a single megacity. We then proportionately distribute the remaining EVs outside the top 30 EV cities according to the vehicle fleet (i.e., grid cells with more vehicles have greater adoption). We estimate the particulate emissions of tire, road, and brake wear using GAINS data for the fraction of total on-road emissions associated with these sources. For simplicity, we assume the EVs that replace ICEVs have the same curb weight and also regenerative braking, that is, we adopt best-case estimates for $r_{TW}E_{s,j,m}^{TW}$, $r_{RW}E_{s,j,m}^{RW}$, and $r_{BW}E_{s,j,m}^{BW}$ of 1.0, 1.0, and 0.0, respectively.

#### 2.5.2. Emissions From EGUs That Power EVs

We calculate the EGU emissions that power EVs as:

(4) $$E_{s,t,j}^{EGU}=ER_{s,t,j}^{EGU}\cdot V_{t,j}$$

where $ER_{s,t,j}^{EGU}$ is the average emission rate (g Wh^−1^ or moles Wh^−1^) of species s for the EGUs in grid cell *x*_*j*_, and *V*_*t,j*_ is the marginal electricity generation (Wh) assigned to grid cell *x*_*j*_. We calculate $ER_{s,t,j}^{EGU}$ by co-locating all EGUs (including emission-free EGUs: solar, hydro, wind, and nuclear) in the Global Power Plant Database (Byers et al., 2018) to a model grid cell. The grid cell average emission rate is calculated as the weighted average of the individual EGU emission rates with the weights equal to the EGUs’ estimated generation. Because our emissions are prescribed on an hourly basis, we are able to improve upon the methods of Schnell et al. (2019) by only allowing solar generation to be used during the day (we assume 7 a.m. to 5 p.m.), effectively increasing nighttime emission rates. EGU emission rates are from the National Bureau of Statistics (2015), which provides rates for NO_x_, SO_2_, total PM, the fraction of total PM that is PM_2.5_, PM_10_, and PM_2.5–10_, and the BC and OC fractions of PM_2.5_ for each province and EGU type. For model-simulated species without EGU emission rates (i.e., VOCs), we assume a conservative scaling factor equal to the lowest emission increase (associated with and only applied to EGU emissions). Since PM_2.5_ emissions are highly speciated in the model emissions (18 species) but the EGU emission rates only provide the fraction of PM_2.5_ that is OC and BC, we set the emission rate of “PMOTHR” (i.e., the unspeciated PM_2.5_ model emission species) equal to the emission rate of PM_2.5_ minus the emission rates of BC and OC. For some experiments (*2010), we set coal-fired EGU emission rates to those in Liu et al. (2015), leaving all other EGU types the same. We scale BC and OC emission rates by the PM_2.5_ rate change between the two datasets. For CO_2_, we use Liu et al. (2015) emission rates for coal-fired EGUs in *2010 experiments, and linearly interpolate to 2013 for the *2015 experiments. For all scenarios, we use U.S. emission rates for gas-fired and oil-fired plants, which are respectively assumed to be 50% of the CO_2_ emission rate of coal-fired EGUs and 743.4 g kWh^−1^ (US DOE, 2016).

#### 2.5.3. Marginal Electricity Generation

The marginal electricity generated at a grid cell *x*_*j*_ required to power EVs at each of K grid cells *x*_*k*_ is:

(5) $$V_{t,j}=\overset{M}{\underset{m=1}{\sum }}\overset{K}{\underset{k=1}{\sum }}\;w_{k,j}^{*}\cdot Q_{t,k,m}$$

where *Q*_*t,k*_ is the electricity requirement for the adopted EVs and $w_{k,j}^{*}$ is a combination of two individual weights, which are functions of distance ($w_{k,j}^{D}$, Equation 6a) and the estimated average electric load ($w_{k,j}^{L}$, Equation 6b).

(6a) $$w_{k,j}^{D}=\{\begin{array}{c} D^{-1}\\ {\left|x_{j}-x_{k}\right|}^{-1}\\ 0 \end{array}\begin{array}{l} \text{if }\left|x_{j}-x_{k}\right|\leq D_{\text{min}}\\ \text{if }D_{\text{min}}<\left|x_{j}-x_{k}\right|\leq D_{\text{max}}\\ \text{ if }\left|x_{j}-x_{k}\right|>D_{\text{max}} \end{array}$$

(6b) $$w_{k,j}^{L}=L\left(x_{j}\right)$$

where *D*_min_ is a minimum distance parameter that prevents a singularity when *x*_*j*_ and *x*_*k*_ are the same grid cell (i.e., $w_{k,j}^{D}=\infty$, which would remap all of the additional electricity required from a grid cell to itself) is set to 100 km. This means that all EGUs within a 100 km radius of the grid cell that requires electricity receive equal distance weighting. *D*_max_ is a maximum distance parameter set to 1,000 km.

#### 2.5.4. Electricity Required to Power EVs

The electricity need for the EVs in grid cell *x*_*k*_ is calculated as:

(7) $$Q_{t,k,m}={\left(1-TL\right)}^{-1}\cdot CE^{-1}\cdot {\left(EV_{eff}\right)}^{-1}\cdot fEV_{j,m}\cdot w^{vkt}VKT_{t,k,m}$$

where *TL* fractional transmission loss (assumed to be 5%), *CE* is the charging efficiency (85%, Huo et al., 2015; Tarroja et al., 2016), *EV*_*eff*_ is the efficiency (km Wh^−1^) of the adopted EV, *fEV*_*j,m*_ as above is the fraction of the ICEVs transitioned to an EV, and *VKT*_*t,k,m*_ is the vehicle kilometers traveled by mode m in grid cell *x*_*k*_ and time *t*. Schnell et al. (2019) used VKT to calculate the electricity need for monthly averaged emissions; however, because our hourly emissions have an imposed diurnal profile associated with anthropogenic activities (e.g., morning rush hour), we make a slight modification (*w*^*vkt*^), which scales the hourly VKT by its inverse (conserving total daily VKT); that is, the diurnal cycle of EV charging (*Q*) and VKT are inversely proportional. The GAINS model provides province-level VKT, which we map onto our 12 km grid in the same way as with the vehicle fleet. *EV*_*eff*_ is experiment dependent.

## 3. Results

### 3.1. Baseline Historic Extreme Pollution Event

**Figure 2.**
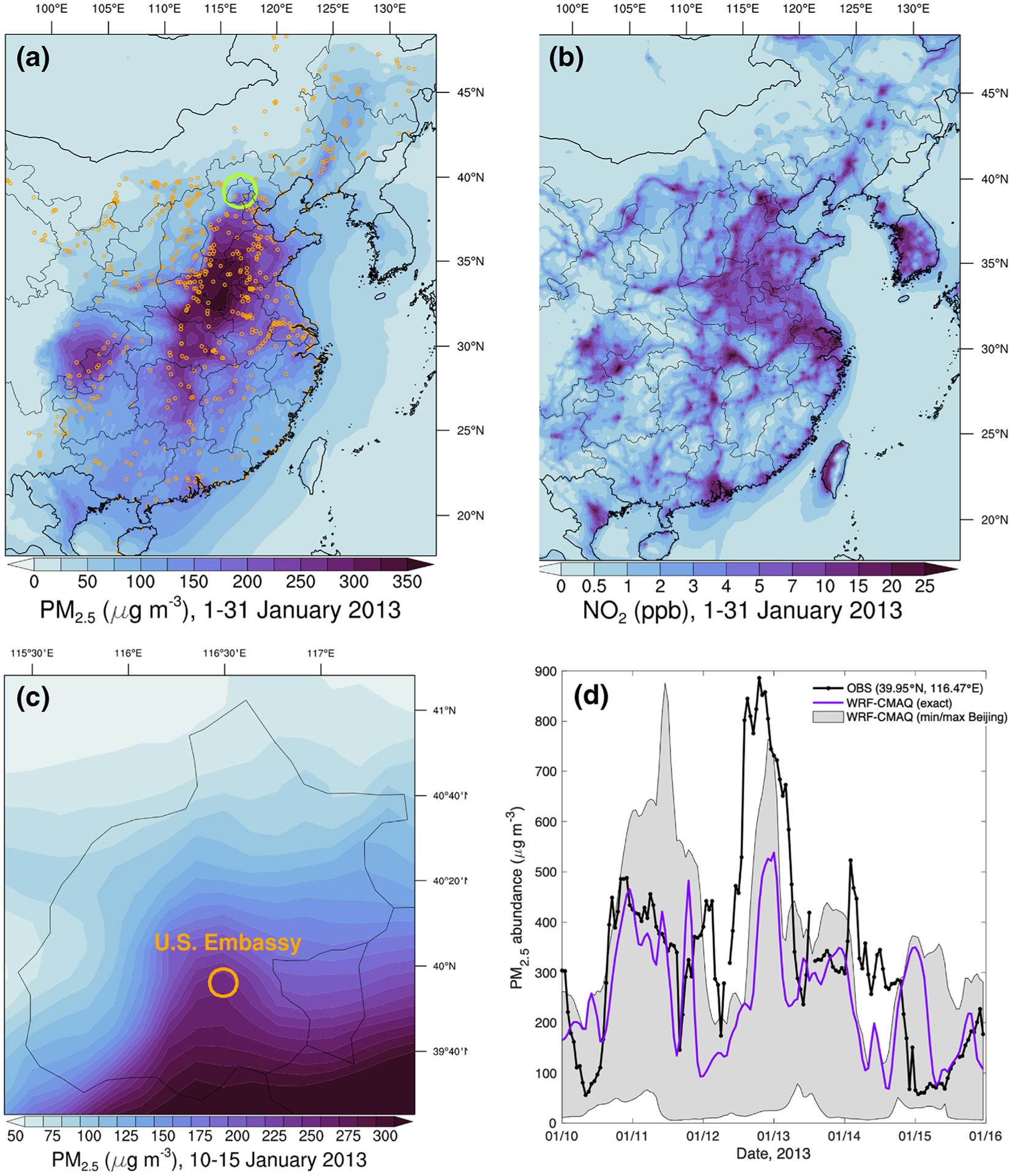
Summary of surface PM_2.5_ for the January 2013 pollution episode over China. (a) Modeled monthly mean PM_2.5_ concentrations in *BASE* over the model domain. The Beijing province is denoted by the green circle, and the orange dots are the location of coal-fired EGUs, (b) as (a) but for NO_2_, (c) Modeled peak episode (10–15 Jan) concentrations over Beijing. (d) Time series of hourly PM_2.5_ abundance observed at the U.S. Embassy (orange in [c]), the model grid cell that contains the Embassy, and the min/max of all grid cells inside Beijing.

Simulated January 2013 average PM_2.5_ concentrations range from ~10 μg m^−3^ over remote areas of China to ~200–350 μg m^−3^ over the North and Central China Plain (NCP) in our baseline historic scenario (BASE; Figure 2a), consistent with observations (Wang et al., 2014). High-population, high-emission, yet geographically diverse megacities of Beijing, Shanghai, and Guangzhou are simulated as pollution hotspots, in addition to the Sichuan basin due to its confining topography. NO_2_, another pollutant with adverse health effects and has potential for reduction through EV adoption, is similarly elevated in megacities, throughout the NCP, and along major highways (Figure 2b). We estimate that across China acute exposure to PM_2.5_ and NO_2_ during the January 2013 episode led to ~32k premature deaths, ~1M hospital admissions, ~8M outpatient visits, ~3M cases of bronchitis, and ~2M cases of asthma, with total economic losses of 14.7B US$ across seven health endpoints (Table S1).

While monthly average PM_2.5_ concentrations were high in many locations during January 2013, the core event and damages were particularly acute in Beijing (e.g., Ferreri et al., 2018; Gao et al., 2015; Sun et al., 2014). During the period of peak PM_2.5_ concentrations (10–15 January), modeled PM_2.5_ across Beijing exhibits a strong north-south gradient, ranging from ~50 μg m^−3^ in the north to over 300 μg m^−3^ in the south (Figure 2b). Observations at the US Embassy recorded concentrations that ranged from 56–886 μg m^−3^, while our model simulates concentrations of 69–539 μg m^−3^ over the Embassy and misses the peak day magnitude (Figure 2d). Across all Beijing grid cells, simulated concentrations range from 5–875 μg m^−3^ (Figure 2d). During the most severe days of the episode (10–15 January, Figures 2c and 2d), we estimate 122 premature deaths from exposure to PM_2.5_ and NO_2_ in Beijing, whereas for the month, we calculate a total of 486 premature deaths, with a total economic impact of over 132M US$ summed across seven health endpoints (Table S1).

### 3.2. Co-Benefits of e-HDV and e-LDV Adoption

**Figure 3.**
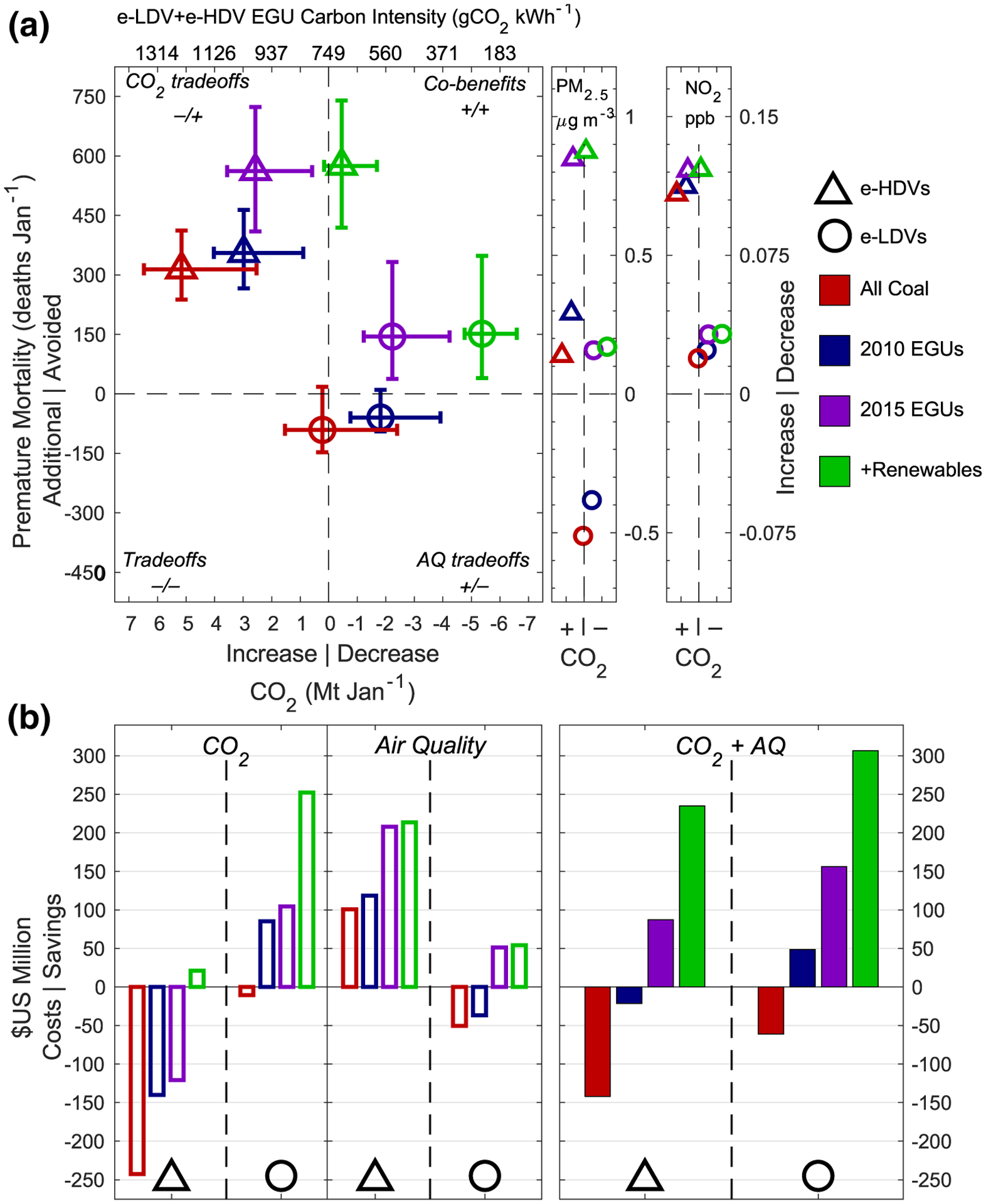
Summary of EV adoption co-benefits and tradeoffs for each e-HDV and e-LDV adoption and power generation scenario. (a) CO_2_ emission reduction (Mt Jan^−1^) and avoided premature mortality (deaths/January). Top *x*-axis provides the carbon intensity of the power sector that correspond with the bottom *x*-axis CO_2_ emission changes for combined e-HDV + e-LDV adoption. Uncertainty bars for CO_2_ are the range of battery efficiencies; for premature mortality, the 95% confidence interval of *β* (exposure-response). Plots at right shows the change in average PM_2.5_ and NO_2_ over grid cells in China. (b) Monetary cost or savings (million US$/January) of EV adoption, shown individually for CO_2_ and health/air quality, and their sum (right, filled bars). EV, Electric vehicle; HDV, heavy-duty vehicle; LDV, light-duty vehicle.

**Figure 4.**
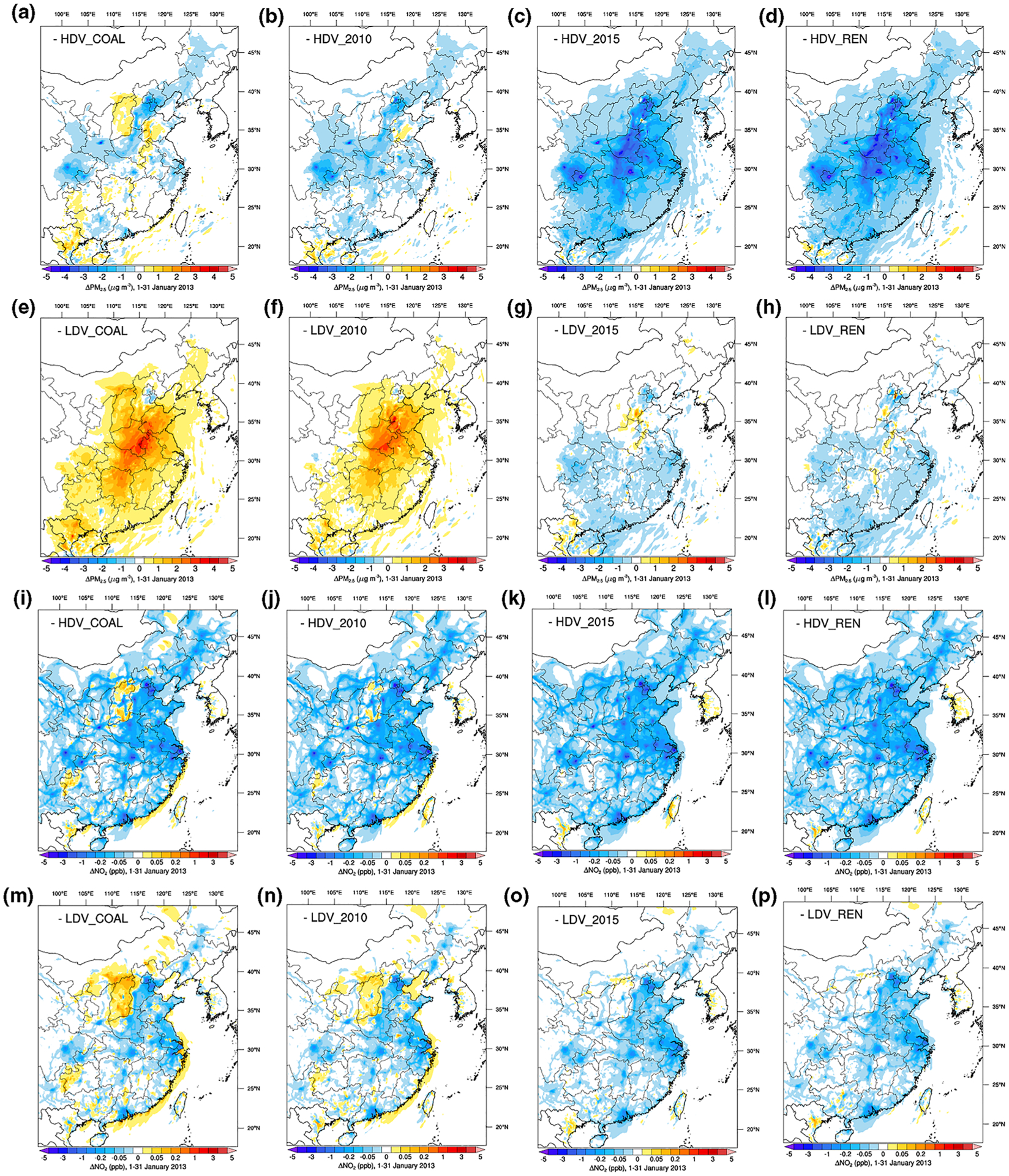
Mean changes PM2.5 (a–h, μg m^3^) and NO_2_ (i–p, ppb) changes for each experiment.

We scrutinize the benefits and tradeoffs of EV policy and implementation decisions on the mitigation of extreme pollution events using metrics that capture emission rates, public health impacts, and/or economic costs (Figure 3 and Table S2). Compared to BASE, a 40% conversion to e-HDVs (1.5M vehicles; Figure 1a) powered by 2015 electricity generation emissions rates (HDV_2015, Table 1) would have avoided 562 (95% CI: 410, 723) premature mortalities in China for the month, following an average PM_2.5_ reduction over China of 0.85 ± 0.82 μg m^−3^ and NO_2_ reduction of 0.58 ± 0.13 parts per billion (ppb) (Figure 4). However, such a transition would increase CO_2_ emissions by 2.6 Mt Jan^−1^ (i.e., a CO_2_-tradeoff). The combined monetary impacts of a CO_2_ increase (valued at $47 per ton CO_2_ (Liang et al., 2019), a loss of 121M US$) with those of seven health endpoints (a savings of 208M US$) largely offset one another such that e-HDV adoption yields a total savings of 87M US$ for the month (Figure 3b).

We compare the co-benefits of e-HDV adoption with a scenario that uses the total electricity demand required for 40% e-HDV adoption to instead electrify a fleet of LDVs (LDV_2015). Because of their substantially smaller per-kilometer electricity requirement, significantly more LDVs are electrified (39.2M; Figure 1b), though coincidently, this is also ~40% of the existing LDV fleet. Air quality improvements for e-LDV adoption are less than for e-HDVs since HDVs contribute more to the on-road emission fraction of both NO_x_ and primary PM_2.5_. e-LDV adoption avoids 145 (95% CI: 38, 333) premature deaths due to a China-averaged PM_2.5_ (NO_2_) reduction of 0.16 ± 0.27 μg m^−3^ (0.02 ± 0.05 ppb). The adoption of e-LDVs avoids ~25% of the number of deaths as e-HDVs, however, e-LDVs dramatically reduce CO_2_ emissions (2.2 Mt Jan^−1^) such that the combined economic impacts of CO_2_ reductions and human health impacts yield a total savings of 156M US$ (Figure 3b).

Province-level CO_2_, PM_2.5_, NO_2_, and associated mortality changes (Figure S4) are expectedly more variable than national averages, but can provide insight into regionally targeted cross-modal EV adoption planning. Similar to previous work (Liang et al., 2019), we find the major metropolitan regions of Beijing-Tianjin-Hebei (BTH), Yangtze River Delta (YRD), and the Pearl River Delta (PRD) (Figure S1) generally experience the largest air quality improvements for both e-LDV and e-HDV adoption scenarios, and thus experience larger reductions in mortality. For HDV_2015, 48% of total avoided mortality occurs in these three regions; for LDV_2015, 59%. Provinces in these regions also contribute 86% of total CO_2_ emission reductions for LDV_2015 while for HDV_2015, only 7 of the 30 provinces in our domain decrease their CO_2_ emissions—three of which are in the major metropolitan regions.

For a month with less extreme meteorology (January 2014), we find that e-HDV health gains are 14% less than those in 2013 due to a smaller reduction in domain-averaged PM_2.5_; for e-LDVs, NO_2_ is reduced similarly to 2013, but the average PM_2.5_ reduction over China is just 0.01 μg m^−3^ (Table S2). Thus, while both e-HDV and e-LDV adoption improve air quality during an extreme meteorological set up, e-LDV adoption results in negligible PM_2.5_ changes during less (un)favorable/extreme meteorological conditions.

Overall, we find that EV-induced PM_2.5_ changes and resultant avoided premature mortality due to acute PM_2.5_ and NO_2_ exposure are modest for this extreme event—a consequence of the small fraction of both primary and precursor PM_2.5_ emissions in the on-road sector (e.g., 13.2% of NO_*x*_ emissions and 3.5% of black carbon emissions in the on-road sector; Table S3). Indeed, in an experiment that removes all on-road emissions over China (NO_TRA), average China NO_2_ decreases by 0.5 ppb, average PM_2.5_ only decreases by 3.2 μg m^−3^, avoiding 1878 premature deaths. Over grid cells where we previously simulated EV adoption the PM_2.5_ (NO_2_) reduction is 4.0 μg m^−3^ (0.8 ppb), and 11.2 μg m^−3^ (3.0 ppb) over Beijing (Figure S5; Table S2). PM_2.5_ reductions are also modest because reduced on-road sector emissions in our EV experiments are offset by increases in power generation emissions, which constitute a much greater fraction of PM_2.5_ (Table S3). Comparatively, removing all emissions associated with power generation (NO_ENE) decreases average PM_2.5_ (NO_2_) by 21.2 μg m^−3^ (0.3 ppb) over China, by 25.1 μg m^−3^ (0.4 ppb) over EV adoption grid cells, and by 32.0 μg m^−3^ (1.2 ppb) over Beijing, leading to 7k+ avoided premature deaths and total health impacts of 3.4B US$ (Figure S5 and Table S2).

### 3.3. CO_2_ Benefits and Tradeoffs

CO_2_ reduction with EV adoption is dependent on battery charging demand. For our EV adoption scenarios to be CO_2_-neutral, the electricity generation mix must have an average CO_2_ emission rate less than ~480 g CO_2_ kWh^−1^ for e-HDVs and ~1,015 g CO_2_ kWh^−1^ for e-LDVs, though these emission rates vary by −11% to +33% over a range of battery efficiency values (i.e., distance-per-charge; Methods). Based on these CO_2_-neutral rates alone, it is clear that e-LDV adoption can achieve net-negative CO_2_ emissions much more readily than e-HDV. Indeed, all e-LDV scenarios can reduce CO_2_ emissions, except in a scenario when e-LDVs have low battery efficiencies and are solely powered by coal-fired EGUs prior to recent emission reductions (LDV_COAL; Figure 3a and Table S2). Conversely, for e-HDV adoption, only in the scenario that assumes a uniform 50% marginal (i.e., the newly required electricity for EVs) carbon-free power generation (HDV_REN; Table 1) are CO_2_ emissions reduced (5.4 Mt yr^−1^). Likewise, the 50% decarbonized scenario for e-LDVs avoids 64.4 Mt yr^−1^ of CO_2_, 37.7 tons more than avoided by LDV_2015.

Since our e-LDV and e-HDV experiments require equivalent electricity demands and both electrify ~40% of their respective fleets, we can compute that an across-the-board 40% adoption of e-LDVs and e-HDVs would require an average CO_2_ emission rate of ~750 g CO_2_ kWh^−1^ (top x-axis in Figure 3a). By combining the CO_2_ emissions changes for e-LDVs plus e-HDVs, we can also assess our results against recent work that electrifies multiple modes simultaneously (Liang et al, 2019; Peng et al., 2018). To be sure, our experiments are not directly comparable since Peng et al. (2018) electrify “all on-road vehicles” and Liang et al. (2019) electrify modes at differing rates (greater for LDVs). In any case, we find that combined e-LDV and e-HDV adoption under the 2015 EGU infrastructure would increase CO_2_ emissions slightly (+0.3 Mt Jan^−1^, −3.7 to +2.3 over the battery efficiency uncertainty range; see Materials and Methods), which aligns with the negligible or modest GHG reductions for cross-modal electrification found previously.

### 3.4. Air Quality Benefits and Tradeoffs

The adoption of 1.5M e-HDVs in China decreases average PM_2.5_ by 0.9 ± 0.8 μg m^−3^ during an extreme pollution episode over the portion of China in our modeling domain (Figure 3a and Table S2). Reductions largely follow the pattern of average PM_2.5_ and occur at nearly all locations except near a cluster of coal plants (orange markers, Figure 2a) on the Shandong and Hebei border, as well as a few grid cells in western Yunnan. For grid cells that include “EV-forward cities” with enhanced EV adoption (see Materials and Methods), decreases are larger (−2.2 ± 0.9 μg m^−3^; Table S2). Percent reductions in PM_2.5_ are more homogeneous, across the country (~2%) with slightly larger reductions in EV-forward cities. NO_2_ changes over China (−0.12 ± 0.26 ppb) follow major roadways and are largest in the major metropolitan regions and EV-forward cities (−1.29 ± 0.76 ppb).

For e-LDV adoption, the magnitude of mean PM_2.5_ changes over all of our averaging locations and all experiments are < 1 μg m^−3^, with increases for LDV_COAL and decreases for all other scenarios (Table S2 and Figure 4). All experiments have domain-average NO_2_ decreases—and e-HDV experiments have 3–5× the decrease as e-LDV. The PM_2.5_ decreases in LDV_2015 occur primarily in the southern half of the domain, with most of the North and Central China Plain (except Beijing and Tianjin) experiencing little change or PM_2.5_ increases (Figure 4).

All e-HDV adoption scenarios result in improvements in air quality and thus decreases in mortality, even when the entirety of the electricity demand is powered by coal-fired EGUs. For e-LDVs, however, only after recent emission reduction policies (i.e., 2015 emission rates) does PM_2.5_ air quality improve, and then only slightly—NO_2_ decreases on average in all experiments (Figure 4). These results align well with previous findings in that cross-modal strategies improve air quality (Liang et al., 2019; Peng et al., 2018), while solely e-LDV adoption would increase air pollutant emissions unless EGU emission rates are reduced below early 2010s levels (Huo et al., 2015); that is, the switch from AQ-tradeoffs to co-benefits for LDV_COAL/2010 to LDV_2015/REN in Figure 3a.

Under scenarios with significantly higher EGU emission rates, the impact of high-emitting coal-fired units becomes more apparent, and the transition from net-positive to net-negative PM_2.5_ air quality benefits occurs for most locations. Under HDV_COAL, many regions see an increase in PM_2.5_ compared to the domain-wide decreases for HDV_2015, although a swath from Beijing to Chengdu and the Shandong province still experiences PM_2.5_ decreases. For LDV_COAL, Beijing, Tianjin, and a few grid cells in Guangxi and Shanghai experience PM_2.5_ decreases, but the majority of the country’s average PM_2.5_ increases by over 2 μg m^−3^.

While the benefits of enhanced renewable power generation are clear in terms of CO_2_ emissions, it has a surprisingly small impact on air quality in our simulations. To be sure, emission rates from the National Bureau of Statistics (2015) that are used in the *_2015 scenarios (Table S4) are significantly lower than those used in recent analyses for “present-day” rates (e.g., Huo et al., 2015), thus the difference in the emission rate of power sector pollutants between 2015 and REN is relatively small compared to the change from 2010 to 2015. For HDV_REN, PM_2.5_ (NO_2_) is reduced by 1.1 μg m^−3^ (0.2 ppb) over EV adoption cells which leads to 575 avoided deaths over China, 1.8× that compared to HDV_2010. For LDVs under 2010 emission rates, although NO_2_ decreases (−0.02 ppb) average PM_2.5_ increases (+0.63 μg m^−3^) resulting in mortality increases (59 deaths incurred), but slightly decreases in the REN scenario (ΔPM_2.5_ = −0.17 μg m^−3^, ΔNO_2_ = −0.03 ppb, and 310 deaths avoided).

**Figure 5.**
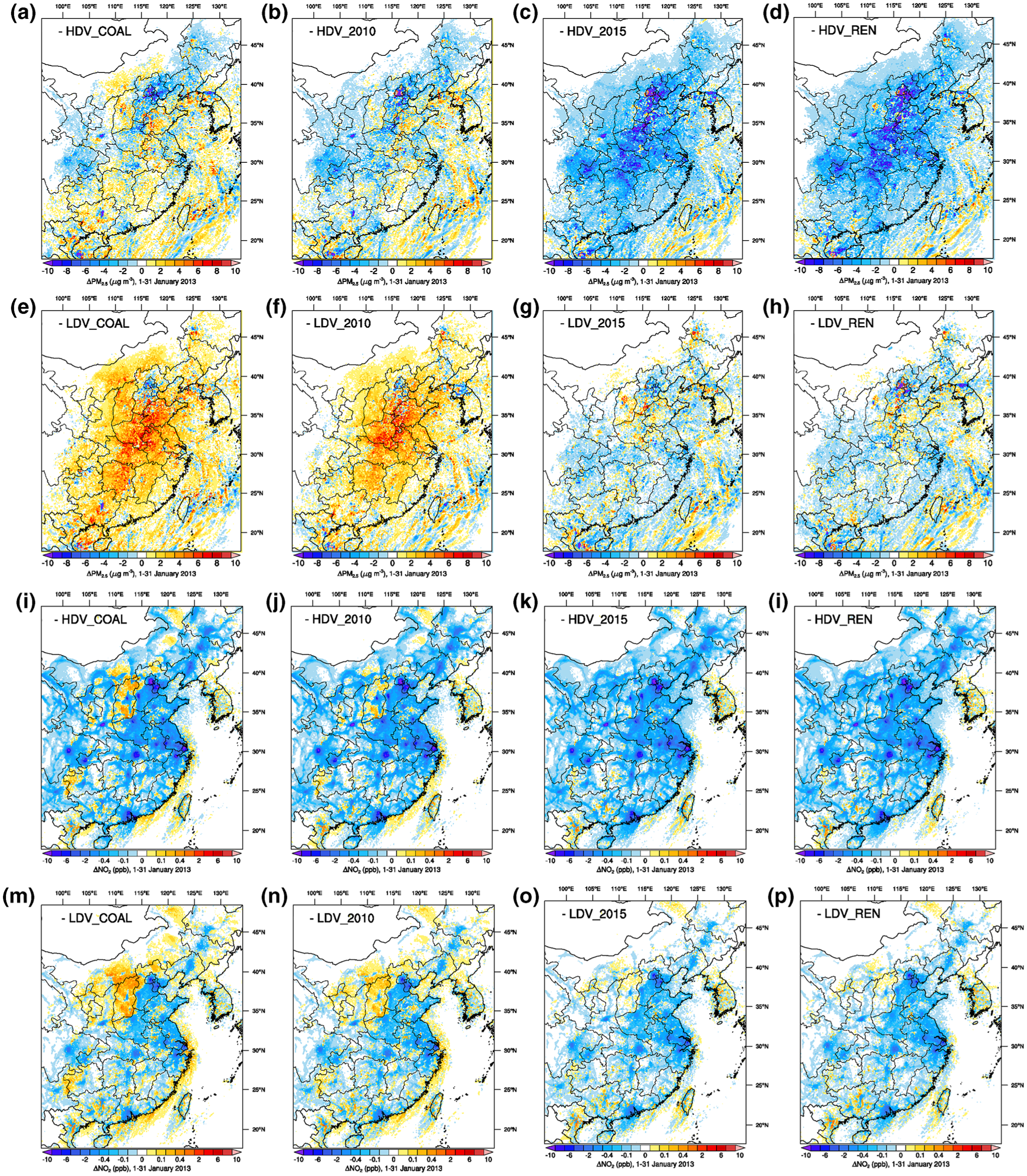
95th percentile PM2.5 (a–h, μg m^3^) and NO_2_ (i–p, ppb) changes for each experiment.

Changes in peak PM_2.5_ (95P) are substantially more heterogeneous (Figure 5 and Table S5), and are predominantly affected by proximity to power generation infrastructure. Under HDV_2015, 95P PM_2.5_ decreases over most of the domain, and are largest in EV-forward cities (−4.5 ± 2.9 μg m^−3^) including a 15.5 μg m^−3^ reduction over Beijing. However, some areas near clusters of coal-fired EGUs in the North China Plain see large increases (>10 μg m^−3^), demonstrating a clear example of a “spillover effect” (Fang et al., 2019); that is, the transfer of urban traffic emissions to rural power generation sites. For LDV_2015 (and further for LDV_2010 and LDV_COAL) PM_2.5_ hotspots near coal-fired EGUs grow in number, extent, and magnitude as they are offset by fewer on-road reductions compared to HDV_2015. The spatial pattern of NO_2_ and PM_2.5_ changes in Figure 5 demonstrates the necessity of a CTM to quantify air quality impacts due to emission changes; that is, NO_2_ is a relatively short-lived pollutant, so changes are largely restricted to emission source regions. PM_2.5_, however, can be transported over much longer distances and also forms secondarily down-wind of source regions.

## 4. Conclusions and Discussion

We have evaluated the potential co-benefits—quantified in terms of avoided acute health impacts and CO_2_ emissions—of hypothetical widespread EV adoption in China during an extreme pollution episode. We have compared our results across vehicle types targeted for electrification (i.e., HDVs vs. LDVs) and demonstrated the sensitivities of the actualized co-benefits of EV adoption to power plant emission rates. Overall, we have shown that the air quality benefits of EV adoption during the January 2013 are modest, with e-HDVs yielding air quality improvements for all power generation scenarios, and e-LDVs requiring emission rate reductions beyond 2010 levels (Figure 3). The reverse is true for CO_2_ reductions: that is, e-LDVs reduce CO_2_ emissions for all power generation scenarios except when powered by all coal-fired electricity generation, while e-HDVs only reduce CO_2_ in a scenario that assumes 50% emission-free marginal electricity generation. Co-benefits are predominately realized in high-population urban centers and industrialized provinces.

A key difference between our work and others examining EV adoption in China is that we only consider acute health impacts and do not consider chronic exposure. Previous annual (i.e., considering chronic exposure) work (Liang et al., 2019) estimated that ~22% of total avoided premature mortality from EV adoption was driven by surface ozone reductions, which we do not consider here since we simulate a cold-season month when ozone is not generally elevated and thus not a health risk.

Future work will be performed an increasingly higher resolution, as emission reductions may not always reduce all pollution types, a result that may otherwise be unresolved by a coarser resolution model. Indeed, The COVID-19 lockdowns of 2020 have revealed the importance of chemical regime in determining how a region may respond to abrupt emission changes (e.g., Diffenbaugh et al., 2020 and references therein). Future work that focuses on the warm season could investigate the urban ozone sensitivity of e-HDV versus e-LDV adoption fractions, as their electrification could impact NO_x_/VOC ratios differently, especially within the urban core. Other improvements are potentially more fundamental, such as designing the modeling framework to maximize use of the results by policymakers and stakeholders, as recent evidence suggests harmonization across studies could be improved (Hess et al., 2020).

China’s chemical landscape is rapidly evolving due to widespread industrialization and substantial pollutant remediation efforts at national and provincial levels. Due to policy-driven changes in energy sector emission rates alone, we find that in less than a decade the air quality benefits of e-LDV adoption switch from a net-negative to a net-positive. Further, air quality will likely continue to improve as the power generation sector decarbonizes and reduces allowable emission rates from fossil fuel-fired EGUs—indeed, an e-LDV purchased in 2013 will be “cleaner” in 2020 than when it was new. Moreover, if reduced fossil fuel-fired energy generation projections are actualized (IEA, 2017), by 2030 the CO_2_ reduction potential from e-LDV adoption will more than double compared to 2015. In any case, recent work suggests that EVs will reduce CO_2_ emissions even without rapid decarbonization of the energy sector in most of the world (Knobloch et al., 2020). In terms of the extreme winter pollution episode mitigation potential of EVs, we find a notable but modest role for widespread EV adoption; however, the long-term benefits are likely at least an order of magnitude greater based on similar pollutant reductions in other EV studies (Liang et al., 2019; Peng et al., 2018). We estimate that acute PM_2.5_ and NO_2_ exposure during the January 2013 extreme pollution episode led to ~32k premature deaths and economic losses of 14.7B US$ across seven health endpoints. Our simulations demonstrate that widespread (40%) e-HDV adoption would reduce just ~1%–2% of these premature deaths, while removal of all on-road transportation sector emissions leads to an ~6% reduction in deaths. Removal of all energy sector emissions however, produces an ~24% drop in premature deaths. Clearly then, carbon- and pollutant-free energy generation is central to the actualization of air quality and climate co-benefits of vehicle electrification in China.

## Supplementary Material

Supplement1

1

## Figures and Tables

**Table 1:** Summary of Modeling Experiments

Scenario name	Scenario description
BASE	Baseline January 2013 scenario
HDV_*	~40% of HDV fleet electrified (1.5 M vehicles)
LDV_*	~40% of LDV fleet electrified (39.2 M vehicles)
*_COAL	EVs powered by coal-fired EGUs using 2010 emission rates
*_2010	EVs powered by EGUs with 2010 emission rates
*_2015	EVs powered by EGUs with 2015 emission rates
*_REN	EVs powerd by 50% renewables
*_2014	Scenario nudged to January 2014 meteorology
NO_TRA	All on-road sector emissions removed from grid cells in China
NO_ENE	All power sector emissions removed from grid cells in China

Abbreviations: EGU, electricity generating unit; EV, Electric vehicle; HDV, heavy-duty vehicle; LDV, light-duty vehicle.

## Key Points:

- Heavy-duty vehicle electrification in China consistently improves air quality regardless of power generation source
- Light-duty vehicle electrification offers less air quality benefits but consistently reduces total CO_2_ emissions
- Power sector emission reductions are central to achieving co-benefits from electric vehicles
