# Supplementary material for: Potential for Electric Vehicle Adoption to Mitigate Extreme Air Quality Events in China
Source: Earths Future. Author manuscript; Available in PMC 2022 Feb 12. (PMC7970456; doi:10.1029/2020ef001788)
Supplement: Supplement1 [file NIHMS1674730-supplement-Supplement1.pdf]

AGU PUBLICATIONS

Earth's Future

Supporting Information for

Potential for electric vehicle adoption to mitigate extreme air quality events in China

J. L. Schnell[1,2], D. R. Peters[3,4], D. C. Wong[5], X. Lu[6], H. Guo[7], H. Zhang[8], P. L. Kinney[9], and D. E. Horton[1]

[1]Department of Earth and Planetary Sciences and Institute for Sustainability and Energy at Northwestern University, Evanston, Illinois, USA

[2]Cooperative Institute for Research in Environmental Sciences at the University of Colorado Boulder NOAA/Global Systems Laboratory, Boulder, Colorado, USA

[3]Program in Environmental Sciences, Northwestern University, Evanston, Illinois, USA

[4]Environmental Defense Fund, Austin, Texas, USA

[5]US Environmental Protection Agency, Research Triangle Park, North Carolina, USA

[6]School of Environment, State Key Joint Laboratory of Environment Simulation and Pollution Control, Tsinghua University, Beijing, China

[7]Department of Earth System Science, University of California Irvine, California, USA

[8]Department of Environmental Science and Engineering, Fudan University, Shanghai, China

[9]Department of Environmental Health, Boston University School of Public Health, Boston, Massachusetts, USA

Corresponding author: Jordan Schnell (jordan.schnell@noaa.gov)

Contents of this file

Figures S1 to S5
Tables S1 to S5

Introduction

This supplementary information includes figures and tables that provide model evaluation, parameter values, and other summary statistics.

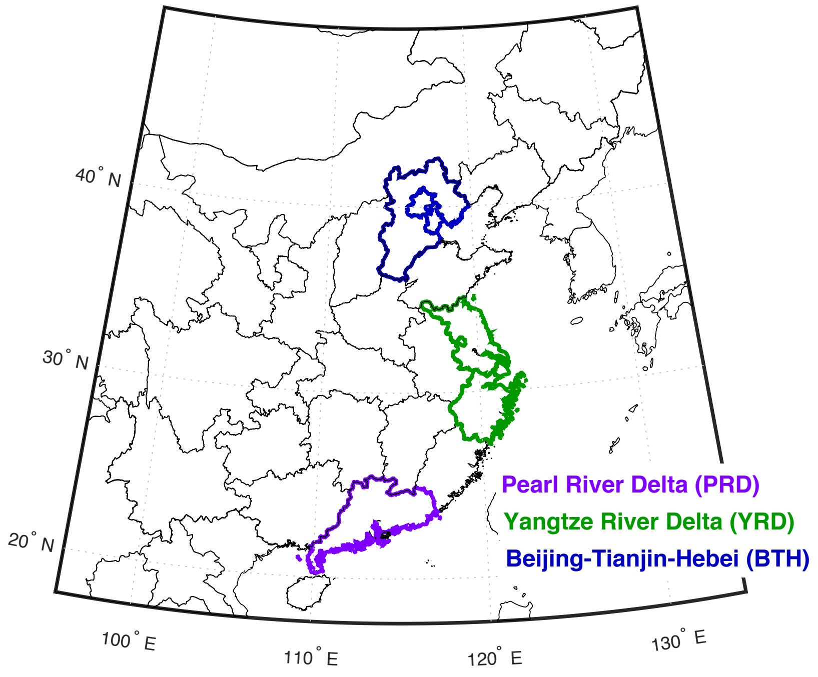

Figure S1. Major industrialized regions.

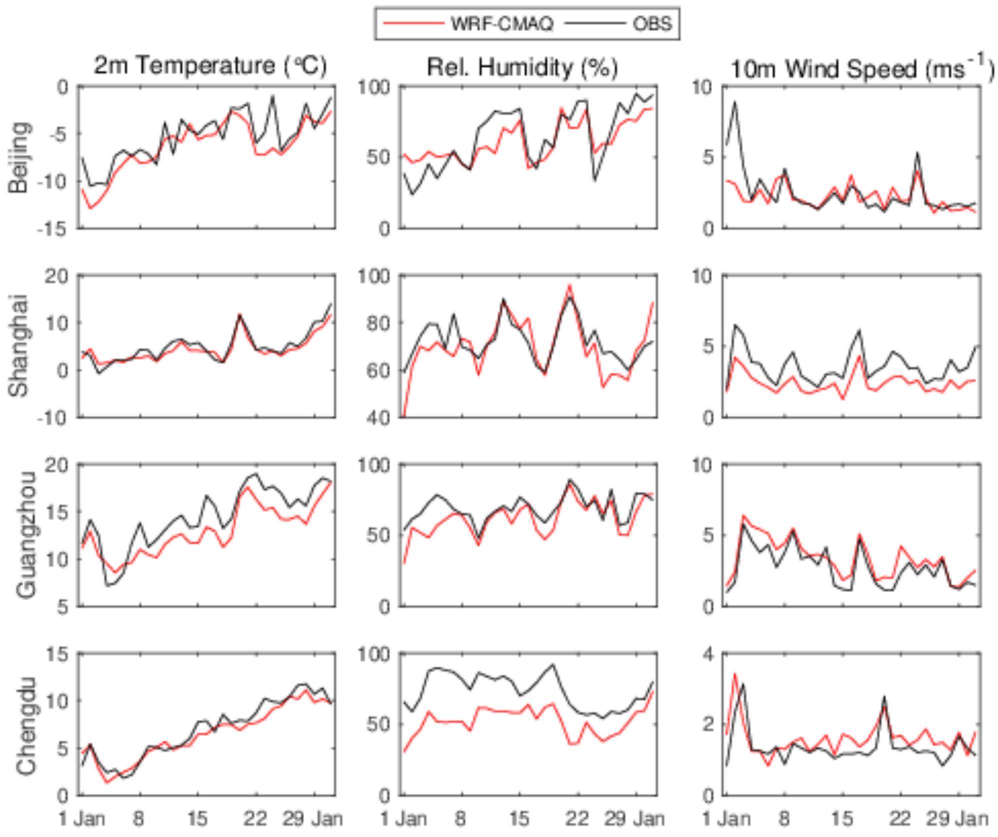

Figure S2. Meteorological comparison of WRF-CMAQ (red) against surface observations sites (NOAA NCEP Integrated Surface Database (https://www.ncdc.noaa.gov/isd/data-access)) nearest the sites where $PM_{2.5}$ is measured.

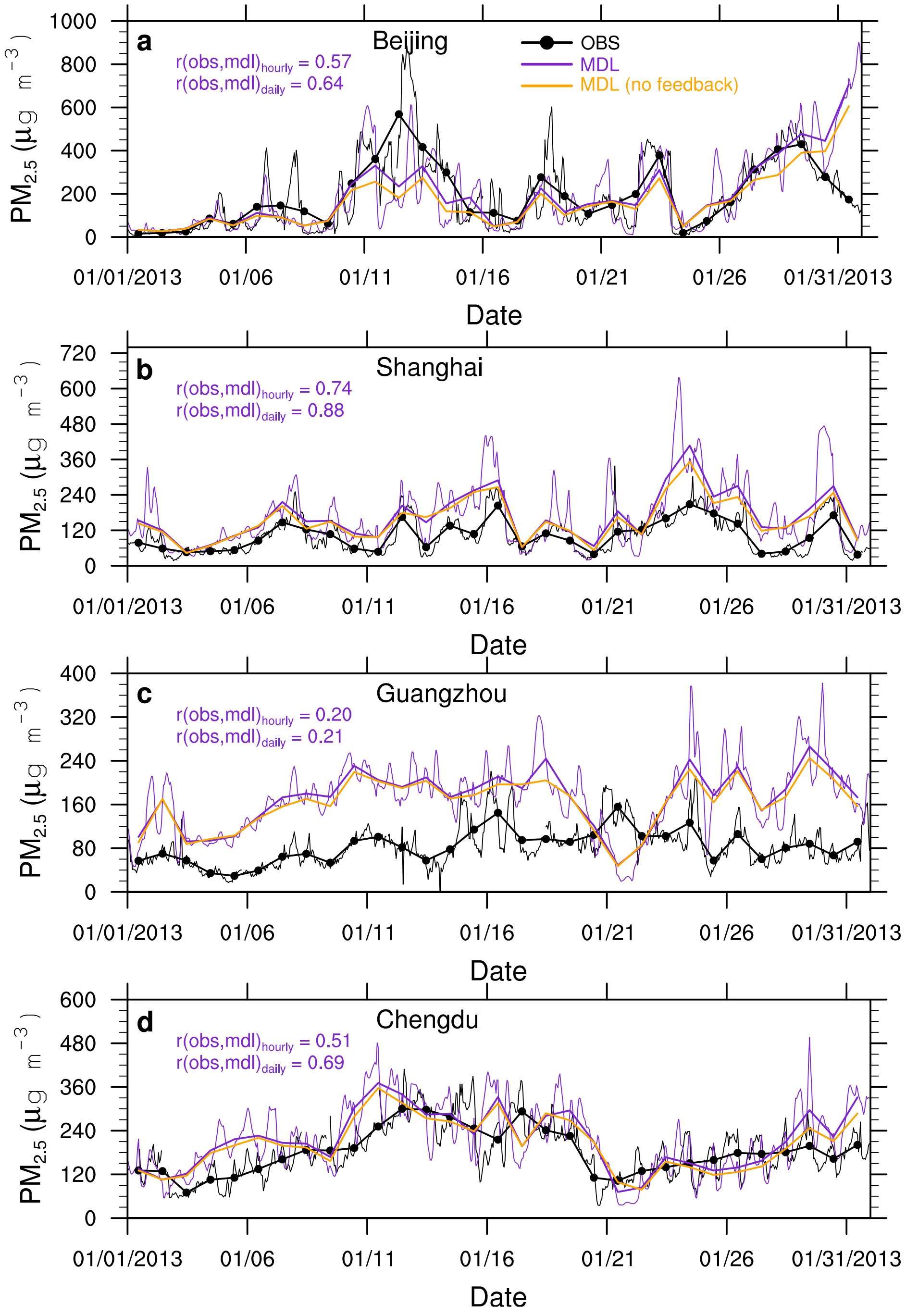

Figure S3. Comparison of WRF-CMAQ to U.S. Embassy observations. Purple lines are the single grid cells over the observation sites for *BASE*, orange is the simulation without shortwave aerosol-radiation feedback.

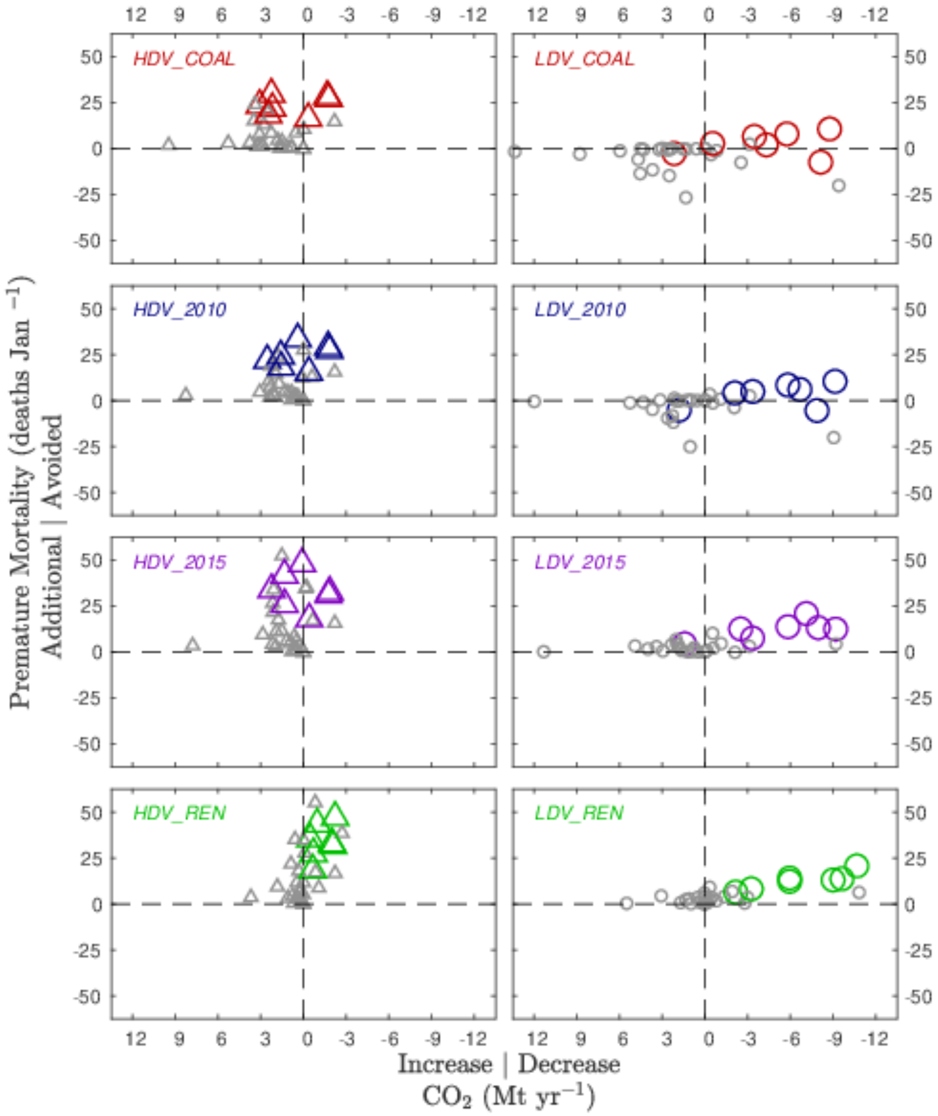

Figure S4. Province co-benefits for each experiment. Colored, large markers are provinces in major industrialized regions (Fig. S1).

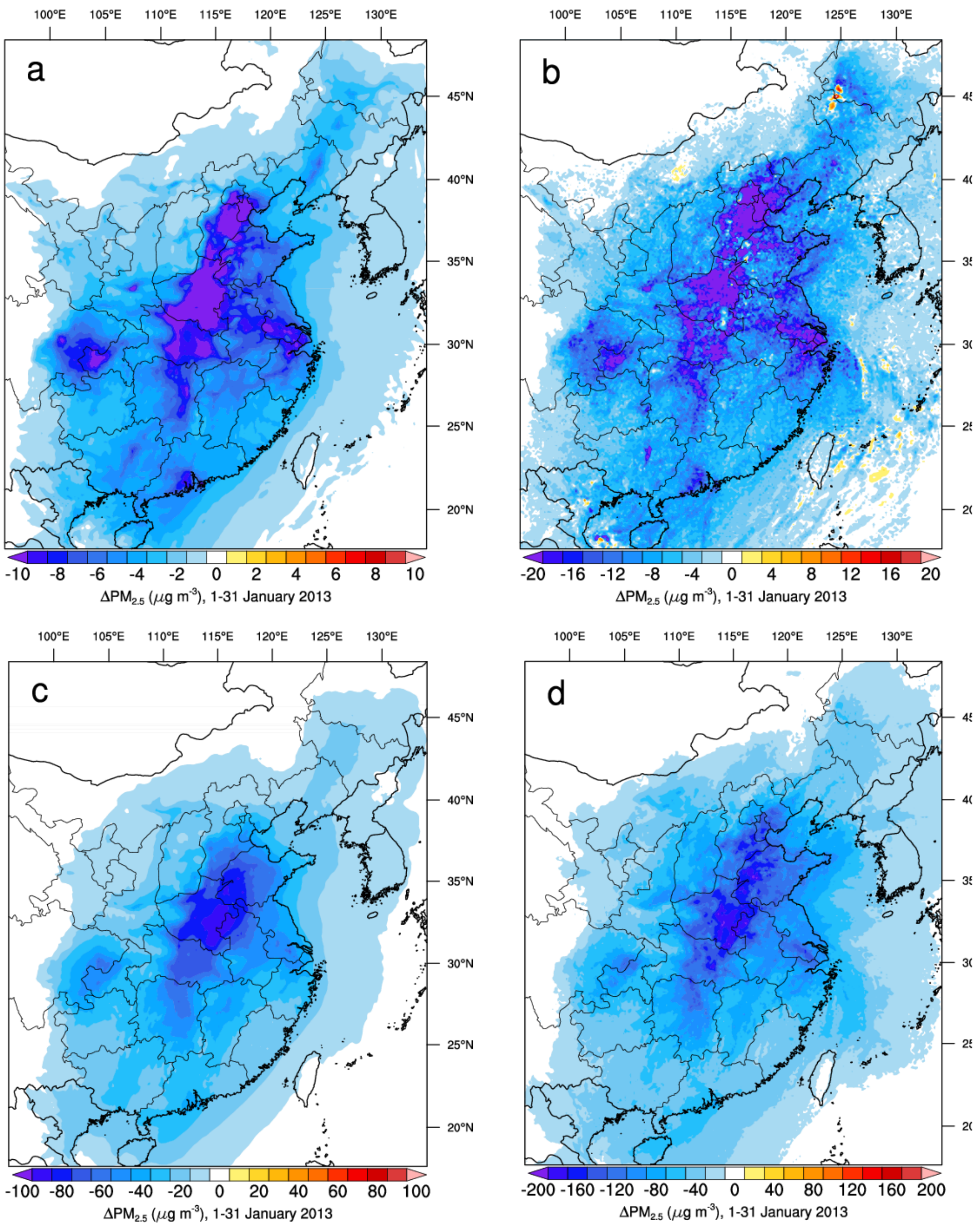

Figure S5. Mean (a, c) and (b, d) 95th percentile changes for (a, b) NO-TRA and (c, d) NO-ENE.

**Health endpoints**	***β* (per 10 μg m^{-3}, 95% CI) (%)**	**References**	**IRs (‰)**	**References**	**Unit Loss (US$/case)**	**# BASE**
Mortality ($PM_{2.5}$)	0.22 (0.15, 0.28)	Chen et al. (2017)	0.022377	BMBPH (2012)	273,513.36	28539
Mortality (NO_2)	0.9 (0.7, 1.2)	Chen et al. (2018)	0.022377	BMBPH (2012)	273,513.36	3013
Respiratory hospital admission	2.0 (1.33, 2.67)	Aunan and Pan (2004), Zhang et al. (2007)	0.051925	BMBPH (2012)	2761.04	499305
Cardiovascular hospital	1.17 (0.5, 1.83)	Aunan and Pan (2004), Zhang et al. (2007)	0.093509	BMBPH (2012)	2761.04	572134
Outpatient visits—internal medicine (15 +)	0.57 (0.32, 0.82)	Xu et al. (1995), Zhang et al. (2007)	2.92083	Zhang et al. (2007)	83.86	8485238
Outpatient visits—pediatrics (0–14)	0.65 (0.23, 1.07)	Xu et al. (1995), Zhang et al. (2007)	0.811925	Zhang et al. (2007)	83.86	250883
Acute bronchitis	9.17 (3.15, 15.18)	Jing et al. (2000), Zhang et al. (2007)	0.140377	Zhang et al. (2007)	407.03	3542312
Asthma	2.1(1.45, 2.74)	Xie et al. (2009)	0.215982	Zhang et al. (2007)	299.61	2159488

Table S1. Parameters, references, and number of cases for *BASE* for seven health endpoints. Parameters and references are a reproduction of Tables 1 and 2 from Gao et al. (2015).

Experiment	$\Delta PM_{2.5}$			ΔNO_2			ΔCO_2	Avoided Mortality	10^6 US$ Saved
	China	EV grid cells	e-forward cities	China	EV grid cells	e-forward cities			
HDV_COAL	-0.14	-0.20	-0.88	-0.11	-0.18	-1.24	62	314	-141
HDV_2010	-0.29	-0.37	-1.05	-0.11	-0.19	-1.25	36	355	-21
HDV_2015	-0.85	-1.04	-2.18	-0.12	-0.20	-1.29	31	562	87
HDV_REN	-0.87	-1.07	-2.25	-0.12	-0.20	-1.29	-5	575	235
LDV_COAL	0.51	0.60	0.66	-0.02	-0.04	-0.38	3	-90	-60
LDV_2010	0.38	0.46	0.51	-0.02	-0.04	-0.39	-22	-59	48
LDV_2015	-0.16	-0.20	-0.62	-0.03	-0.05	-0.43	-27	145	155
LDV_REN	-0.17	-0.21	-0.61	-0.03	-0.06	-0.43	-64	152	306
HDV_2015 (2014m)	-0.56	-0.70	-1.70	-0.13	-0.21	-1.33	31	485	54
LDV_2015 (2014m)	-0.01	-0.04	-0.46	-0.04	-0.06	-0.46	-27	108	137
NO_TRA	-3.21	-3.95	-7.42	-0.46	-0.75	-2.97	n/a	1877	703
NO_ENE	-21.15	-25.13	-40.85	-0.30	-0.39	-1.15	n/a	7687	3321
[a]Health impact only									

Table S2. Summary of EV co-benefits: mean changes in $PM_{2.5}$ (μg m^{-3}) and NO_2 (ppb), CO_2 emission changes (Mt yr^{-1}), avoided mortality (deaths/January, and economic valuation (CO_2 + seven health endpoints) for each experiment. "2014m" refers to 2014 meteorology.

	NO_x	SO_2	BC	PMOTHR	CO	HCHO
On-road	13.3	0.2	3.5	0.2	9.7	25.0
Energy	35.3	27.0	1.4	11.4	0.8	0.0

Table S3. Fraction of total emissions in the on-road and energy generation sectors.

Experiment	CO_2	SO_2	NO_x	$PM_{2.5}$
COAL/2010	905.6	2.48	2.67	0.27
2015/REN	861.0	0.42	0.35	0.10

Table S4. Average coal-fired EGU emission rates.

Experiment	$\Delta PM_{2.5}$			ΔNO_2		
	China	EV grid cells	e-forward cities	China	EV grid cells	e-forward cities
HDV_COAL	-0.25	-1.53	-1.52	-0.37	-4.21	-4.26
HDV_2010	-0.62	-2.19	-2.20	-0.38	-4.23	-4.28
HDV_2015	-1.86	-4.58	-4.53	-0.40	-4.29	-4.35
HDV_REN	-1.94	-4.79	-4.73	-0.41	-4.28	-4.34
LDV_COAL	1.25	1.72	1.57	-0.08	-1.32	-1.46
LDV_2010	0.96	1.47	1.26	-0.10	-1.33	-1.47
LDV_2015	-0.26	-1.07	-1.07	-0.12	-1.40	-1.54
LDV_REN	-0.32	-1.26	-1.42	-0.12	-1.41	-1.56
HDV_CUR (2014m)	-1.49	-4.37	-4.33	-0.42	-4.28	-4.37
LDV_CUR (2014m)	-0.08	-0.94	-1.11	-0.13	-1.41	-1.59
NO_TRA	-6.87	-15.20	-15.38	-1.41	-8.62	-9.62
NO_ENE	-48.63	-91.84	-88.70	-0.81	-1.86	-3.62

Table S5. 95th percentile $PM_{2.5}$ changes ($\mu g\ m^{-3}$) for each experiment. “2014m” refers to 2014 meteorology
