# Supplementary material for: Potential for Electric Vehicle Adoption to Mitigate Extreme Air Quality Events in China
Source: Earths Future. Author manuscript; Available in PMC 2022 Feb 12. (PMC7970456; doi:10.1029/2020ef001788)
Supplement: 1 [file NIHMS1674730-supplement-1.pdf]

References From the Supporting Information

Aunan, K., & Pan, X.-C. (2004). Exposure–response functions for health effects of ambient air pollution applicable for China–A meta-analysis. *Science of the Total Environment*, *329*, 3–16. https://doi.org/10.1016/j.scitotenv.2004.03.008

Beijing Municipal Bureau of Public Health (BMBPH) (2012). *Beijing Health Yearbook*. Beijing, China: Beijing Science and Technology Press.

Jing, L., et al. (2000). Relationship between air pollution and acute and chronic respiratory diseases in Benxi. *Journal of Environmental Health*, *17*, 268–270.

Xie, P., Liu, X., Liu, Z., Li, T., & Bai, Y. (2009). Exposure–response functions for health effects of ambient particulate matter pollution applicable for China. *China Environmental Science*, *29*, 1034–1040.

Xu, X., Dockery, D. W., Christiani, D. C., Li, B., & Huang, H. (1995). Association of air pollution with hospital outpatient visits in Beijing. *Archives of Environmental Health*, *50*, 214–220. https://doi.org/10.1080/00039896.1995.9940390

Zhang, M., Song, Y., & Cai, X. (2007). A health-based assessment of particulate air pollution in urban areas of Beijing in 2000–2004. *Science of the Total Environment*, *376*, 100–108. https://doi.org/10.1016/j.scitotenv.2007.01.085
